# Case Report: Identification of a *de novo* missense variant in the N-terminal zinc-finger domain of *ZEB2* in a patient presenting with neurodevelopmental delay and recurrent pulmonary infections

**DOI:** 10.3389/fgene.2026.1778727

**Published:** 2026-02-18

**Authors:** Jing Chen, Xiaodong Jiang, Wei Su, Jingjing Li, Shuang Chen, Ke Xu, Juxian Teng, Weihong Zhang, Hongmin Zhu

**Affiliations:** 1 Department of Rehabilitation Medicine, Wuhan Children’s Hospital, Tongji Medical College, Huazhong University of Science and Technology, Wuhan, China; 2 Pediatric Gastroenterology, Wuhan Children’s Hospital, Tongji Medical College, Huazhong University of Science and Technology, Wuhan, China; 3 Chigene (Beijing) Translational Medical Research Center Co., Ltd., Beijing, China; 4 Department of Pediatric Hematology and Oncology Center, Wuhan Children’s Hospital, Tongji Medical College, Huazhong University of Science and Technology, Wuhan, China

**Keywords:** developmental delay, novel clinical syndrome, N-terminal zinc-fingerdomain, recurrent pneumonia, ZEB2, zinc supplementation

## Abstract

**Background:**

Heterozygous variants in the *ZEB2* gene are known to cause Mowat–Wilson syndrome (MWS). The classical clinical spectrum of MWS includes characteristic facial features, intellectual disability, epilepsy, Hirschsprung disease (HSCR), and various congenital malformations. Reported pathogenic variants have predominantly been truncating variants or missense variants involving the C-terminal zinc-finger domain. To date, no disease-causing missense variant affecting the N-terminal zinc-finger domain has been documented.

**Case presentation:**

We report a 3-year-old boy presenting with characteristic facial features, global developmental delay, and recurrent respiratory tract infections. Trio-based exome sequencing identified a *de novo* heterozygous missense variant in *ZEB2,* c.652C>T (p. Arg218Trp), located within the N-terminal zinc-finger domain. The patient exhibited a phenotype distinct from classical MWS, characterized by atypical facial dysmorphisms (including an elongated face, midface hypoplasia/depression, frontal bossing, esotropia, and hypertelorism), global developmental delay, and recurrent respiratory infections. Following comprehensive rehabilitation therapy (motor, cognitive, and language training) combined with oral zinc supplementation (elemental zinc 5 mg/day, approximately 0.3 mg/kg), the patient showed a marked reduction in respiratory infections and normalization of immune parameters after 12 months of treatment.

**Conclusion:**

This report describes a patient with a *de novo* missense variant in the N-terminal zinc-finger domain of *ZEB2* who presented with neurodevelopmental delay, atypical facial features, and recurrent respiratory infections, alongside a reduction in infection frequency during zinc supplementation. The variant is classified as likely pathogenic, and these observations expand the phenotypic variability potentially associated with *ZEB2* variants. Additional cases and functional studies are required to confirm any causal link between the variant, the observed phenotype, and the effects of zinc supplementation.

## 1 Introduction

The *ZEB2* gene encodes zinc-finger E-box binding homeobox 2, a member of the C2H2-type zinc-finger and ZF homeobox protein families located on chromosome 2q22.3. *ZEB2* functions as a DNA-binding transcriptional repressor that interacts with activated *SMAD* proteins and *TGF-β* signaling mediators, and it associates with the nucleosome remodeling and histone deacetylase (NuRD) complex. However, *ZEB2* can also act as an activator depending on the availability of specific cofactors and the cellular context (Epifanova et al., 2019). Through these interactions*,ZEB2* plays essential roles in multiple processes of embryonic developmental processes, and its loss of function can lead to multisystem abnormalities (Van de Putte et al., 2007).Mowat–Wilson syndrome (MWS, OMIM: 235730) is a rare autosomal dominant disorder caused by pathogenic variants in *ZEB2*, most of which arise *de novo*. The syndrome was first described in 1998 (Mowat et al., 1998).Classic clinical manifestations include characteristic facial features, Hirschsprung disease, microcephaly, intellectual disability, epilepsy, hypertelorism, and a spectrum of congenital malformations such as congenital heart disease and asplenia (Cordelli et al., 2021). Zinc, an essential trace element in humans, plays a critical role in maintaining respiratory and neurological function (Wessels et al., 2020; Vali et al., 2025). Growing evidence suggests that zinc exerts antiviral effects by inhibiting viral RNA polymerases (such as that of SARS-CoV-2) and modulating interferon responses (te Velthuis et al., 2010). Zinc also participates in neurodevelopment and neuronal differentiation, cell survival and apoptosis, neurotransmission and synaptic plasticity, as well as the antioxidant defense of the nervous system (Vali et al., 2025; Lewicka et al., 2017; Li et al., 2022). Consequently, zinc supplementation has shown therapeutic promise in certain genetic disorders such as acrodermatitis enteropathica (Bellini et al., 2024) and Wilson disease (Li et al., 2024).

Here, we report a patient with a *de novo* missense variant located within the N-terminal zinc-finger domain of *ZEB2* presenting with neurodevelopmental delay and recurrent pulmonary infections. Following zinc supplementation, a reduction in infection frequency was observed. These observations raise the possibility that the phenotype may relate to dysfunction in the N-terminal zinc-finger domain, though this remains speculative in the absence of functional assays.

## 2 Materials and methods

### 2.1 Ethical statement

This study was approved by the Ethics Committee of Wuhan Children’s Hospital (Grant No. 2022R043-E01). Written informed consent was obtained from the patient’s family for the use of clinical data, genetic analysis, and publication of facial photographs.

### 2.2 Case presentation

The patient was a 3-year-old boy born to healthy, non-consanguineous parents. He was conceived *via in vitro* fertilization and was the firstborn of a twin pregnancy. He was delivered at 28+^5^ weeks of gestation, with a birth weight of 1.3 kg, and classified as appropriate for gestational age (AGA) based on standard growth charts (Fenton and Kim, 2013). Preterm birth was due to premature rupture of membranes. After birth, he required prolonged hospitalization for 5 months in the neonatal unit and intensive care units of multiple hospitals due to neonatal respiratory distress syndrome, bronchopulmonary dysplasia, and severe pneumonia. His dizygotic twin sister died at 5 months of age from severe pneumonia and did not exhibit features suggestive of MWS. No detailed genetic testing was performed on the deceased twin. At 10 months of age, he underwent surgical treatment for bilateral inguinal hernia and bilateral hydrocele. His developmental milestones were markedly delayed compared with peers: stable head control at 10 months, independent sitting at 20 months, and first words (“mama”) at 18 months. Before the age of 2 years, he experienced chronic constipation, with bowel movements occurring every 4–5 days, and feeding difficulties were notable during infancy. Before the age of 3 years, he experienced four to six episodes of respiratory tract infection per year, at intervals of 1–3 months; during autumn and winter, infections occurred twice within a single month. Independent walking was achieved at 3 years and 9 months. Repeated immune function assessments, including Treg cell analysis, revealed persistent abnormalities. Specifically, CD3^+^CD8^+^ T-cell proportions were reduced at 12.14% (reference range: 20%–38.53%), with an absolute count of 282 cells/μL (reference range: 314–2080 cells/μL).

**FIGURE 1:**
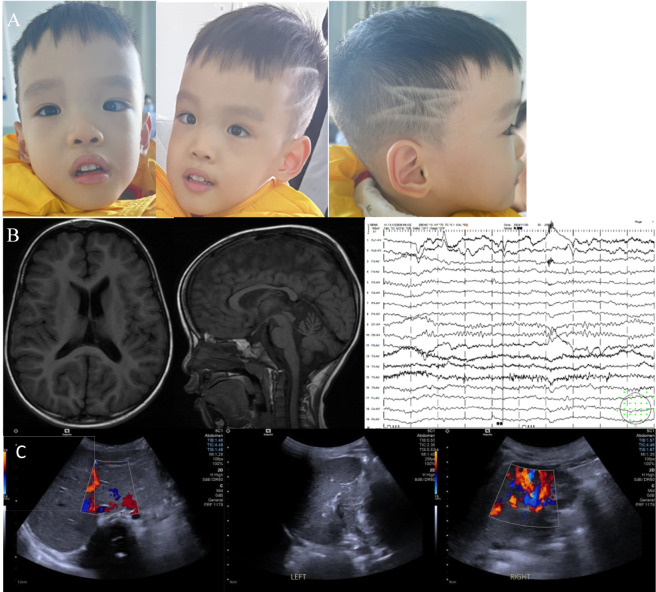
Clinical and imaging findings of the patient. **(A)** Facial photographs at 3 years of age showing an elongated face, midface hypoplasia, frontal bossing, hypertelorism, arched eyebrows, low-set ears, esotropia, and scaphocephaly. Head circumference was 37 cm (<third percentile). **(B)** Left and middle panels: Electroencephalogram showing normal background activity. Right panel: Non-contrast brain MRI at 3 years of age revealing no significant abnormal signal in the bilateral frontal, parietal, temporal, or occipital lobes. No widening of sulci, fissures, or cisterns was observed, and the ventricular system was not enlarged. The cerebellum and brainstem appeared normal. **(C)** Left and middle panels: Abdominal ultrasound at 4 years of age showing normal liver morphology. Right panel: Renal ultrasound demonstrated bilaterally reduced renal longitudinal diameters compared with age-matched norms. Left kidney: 6.0 cm × 2.5 cm (anteroposterior diameter) × 2.7 cm; right kidney: 6.0 cm × 2.6 cm (anteroposterior diameter) × 2.5 cm.

Upon admission at 3 years of age, the patient’s clinical assessment revealed a weight of 11.5 kg (<P3), height of 92 cm (P10–P25), and head circumference of 37 cm (<P3). Characteristic facial features included frontal bossing, hypertelorism, midface hypoplasia, arched eyebrows, low-set ears, and esotropia. Developmentally, he exhibited delayed responsiveness and was able to speak only a few single words, without the ability to form phrases or sentences. Neuromotor examination indicated stable head control and independent sitting; he was able to crawl in a prone position but could not crawl on hands and knees. He could stand with support but was unable to stand or walk independently. Muscle tone was decreased in all four limbs. No stereotypic behaviors or seizure episodes were observed (Figure 1A, facial features). Cardiac ultrasound showed a patent foramen ovale was noted at birth, and follow-up echocardiography at 3 years of age demonstrated normal cardiac anatomy. Brain MRI showed no structural abnormalities, with normal ventricular system, corpus callosum, and posterior fossa domains (Figure 1B). Routine EEG showed normal background activity (Figure 1B). Abdominal ultrasound showed normal liver and spleen. Renal ultrasound demonstrated slightly reduced bilateral renal longitudinal diameters compared with age-matched norms (Figure 1C). Gesell Developmental Schedules indicated the following developmental quotients: adaptive behavior DQ 53, gross motor DQ 24, fine motor DQ 56, language DQ 37, and personal-social DQ 42. Laboratory evaluations, including liver and kidney function, myocardial enzyme levels, electrolytes, immunoglobulin levels (IgA, IgG, IgM), were all within normal limits. Serum zinc concentration was also normal at 75.3 μmol/L (reference range:73∼220 μmol/L).

Before age 3 years, the patient experienced four to six respiratory tract infections per year (intervals of 1–3 months, more frequent in autumn/winter), typically presenting as pneumonia with fever and cough, requiring intravenous antibiotics and showing bronchopneumonia with linear opacities on imaging.

**FIGURE 2:**
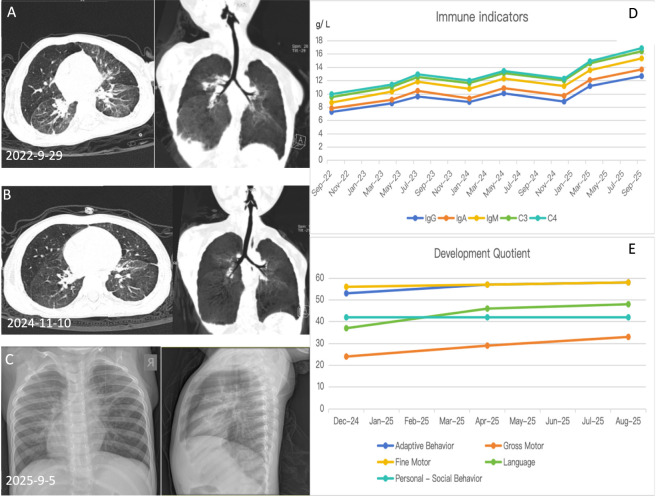
Serial chest imaging and immune parameter trends following zinc supplementation. **(A)** Chest CT (29 September 2022, Lung window) showing increased bilateral lung markings, band-like dense opacities in both lungs, and focal pleural reactions. (Note: Early respiratory infections may be partly attributable to bronchopulmonary dysplasia sequelae). **(B)** Chest CT (10 November 2024, Lung window) showing prominent bilateral lung markings, fibrotic linear opacities in both lungs, and localized patchy areas of increased density, along with focal pleural reactions. **(C)** Chest X-ray (5 September 2025) showing prominent bilateral lung markings with linear and patchy hazy opacities. **(D)** Serial immunoglobulin measurements (IgG, IgA, IgM, C4, C3) remained within normal ranges and demonstrated a gradual upward trend. **(E)** Gesell Developmental Schedules showed overall improvement in developmental quotients compared with earlier assessments.

Considering reported benefits of zinc in recurrent respiratory tract infections (Lassi et al., 2016; Martinez-Estevez et al., 2016; Paediatricemergency and Departmentofpharmacology, 2016), oral zinc supplementation (elemental zinc 5 mg/day≈ 0.3 mg/kg) was initiated at age 3 years for 12 months, alongside continued rehabilitation. During follow-up, infection frequency markedly decreased (intervals of 3–6months; only three episodes in the subsequent year), infections became milder (mainly bronchitis without fever), and most resolved without antibiotics. Repeated immune evaluations showed gradual increases in immunoglobulin levels consistent with age-related norms (Mayo Clinic, 2025) (Figure 2). At age 4 years, gross motor function and language have improved substantially: the patient walks short distances indoors and produces simple 4-5word sentences.

### 2.3 Genetic tests and variants interpretation

Trio whole-exome sequencing (WES) and subsequent bioinformatic analyses were performed by Chigene Ltd. (Beijing, China). Chromosomal G-banding and array comparative genomic hybridization (CGH) were performed and showed normal results. The pathogenicity of candidate variants was evaluated in accordance with the guidelines of the American College of Medical Genetics and Genomics (ACMG) (Miller et al., 2023) and the recommendations of the Sequence Variant Interpretation Working Group (SVIWG, https://www.clinicalgenome.org/working-groups/sequence-variant-interpretation/). Variant interpretation for the ZEB2 gene was based on the reference transcript NM_014795.3.

**FIGURE 3:**
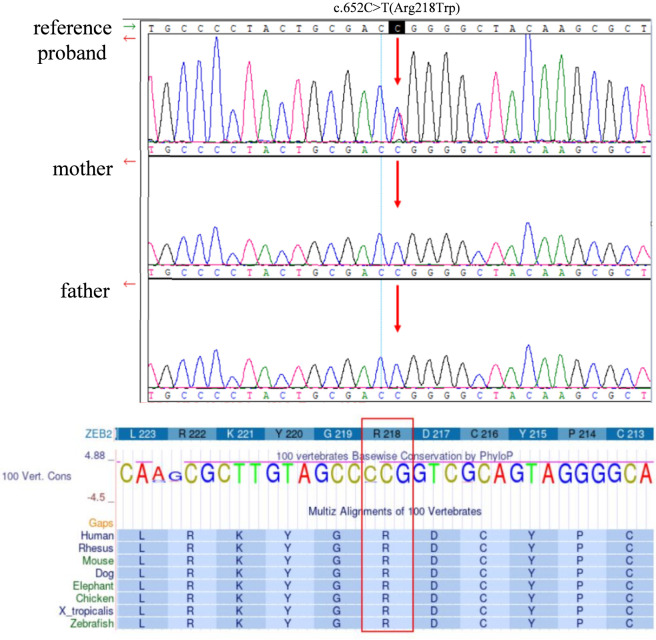
Genetic confirmation and conservation analysis of the *ZEB2* variant. Sanger sequencing confirmed the *de novo ZEB2* c.652C>T (p. Arg218Trp) variant in the patient. (p. Arg218Trp) is highly evolutionarily conserved across species.

Trio WES identified a *de novo ZEB2* variant, c.652C>T (p. Arg218Trp), in the patient. This variant affects a highly evolutionarily conserved amino acid residue. In silico predictions include SIFT: deleterious (score 0.00), PolyPhen-2: probably damaging (score 0.99), and CADD: 35. According to ACMG guidelines, the c.652C>T variant was classified as likely pathogenic, due to *de novo* occurrence (PS2), absence from population databases (PM2), and multiple lines of computational evidence supporting a deleterious effect (PP3). Conservation analysis confirmed strong evolutionary conservation at Arg218 residue (Figure 3).

## 3 Discussion

In the present case, we identified a *de novo ZEB2* missense mutation c.652C>T (p.Arg218Trp),in a child with global developmental delay, supporting diagnosis of MWS. The patient exhibited global developmental delay and atypical facial features (elongated face, midface hypoplasia, frontal bossing, hypertelorism, arched eyebrows, low-set ears, esotropia, scaphocephaly), some of which may be influenced by prematurity. He lacked hallmark features of classical MWS, such as Hirschsprung disease (HSCR), epilepsy, or structural congenital heart defects, cup-shaped ears,or sparse eyebrow. Moreover, although his immune evaluations repeatedly showed low CD3^+^CD8^+^ T-cell proportions, immunoglobulin levels and serum zinc concentrations remained normal, pointing toward a cellular immune shift rather than an antibody deficiency. Combined with his relatively mild infection history and normal splenic development, this immunological phenotype contrasts sharply with previously reported MWS cases, which often present with more severe immune dysfunction, including asplenia or hyposplenia and susceptibility to pneumococcal infections, sepsis, meningitis, or immunoglobulin deficiencies (Frith et al., 2021).Additionally, the scaphocephaly and elongated facial configuration observed in this case have not been documented among previously reported MWS phenotypes (Ghoumid et al., 2013). This unique clinical constellation expands the phenotypic variability associated with *ZEB2-related* disorders and suggests that N-terminal zinc-finger (N-ZF) variants may define a distinguishable, milder phenotypic spectrum. The present case therefore provides valuable insight into ZEB2 function and broadens the clinical spectrum associated with its pathogenic variants.

The *ZEB2* protein contains conserved N- and C-terminal zinc-finger domains. The N-terminal domain (N-ZF) binds E-box motifs and recruits the NuRD corepressor complex, playing roles in neural crest development, neurodevelopment, and immune cell differentiation (Remacle et al., 1999). Animal studies indicate that disruption of the N-ZF domain can reduce forebrain volume and peripheral CD8^+^ T-cell levels (Dai et al., 2024; Benito-Kwiecinski et al., 2021). The p.Arg218Trp variant affects a highly conserved residue in this domain, potentially impairing its DNA-binding or zinc-coordination function, which may contribute to the observed neurodevelopmental delay and recurrent infections in this patient (Hossain et al., 2025). Based on the known roles of the N-ZF domain in immune cell regulation and neurodevelopment, one might hypothesize that dysfunction in this domain could contribute to phenotypes like recurrent infections and developmental delay.

**FIGURE 4:**
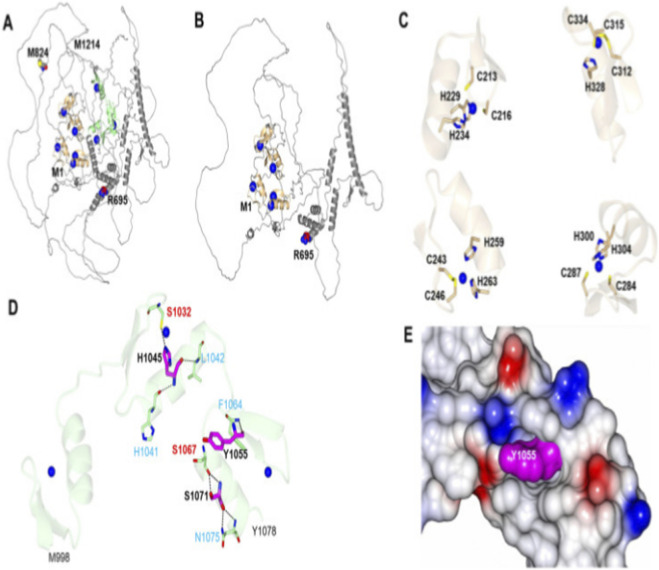
Schematic of zinc-coordination residues within the N-terminal zinc-finger (N‐ZF) domain of ZEB2. **(A,B)** Ribbon diagrams highlighting key residues (M824, M1214, R695, and M1) with colored spheres marking zinc‐binding sites. **(C)** Close-up views of four protein regions illustrating metal‐coordination sites with labeled residues. **(D)** Detailed ribbon diagram showing residues S1032, S1067, S1071, Y1055, H1041, H1045, L1042, F1064, and N1075, color-coded by functional region. **(E)** Molecular surface representation of a protein pocket with residue Y1055 highlighted in magenta, surrounded by colored regions indicating molecular properties.

The N-ZF domain is enriched in histidine and cysteine residues, which coordinate with zinc ions to form a stable folded domain. Zinc binding is essential for maintaining the unique three-dimensional conformation of the N-ZF domain, thereby preserving its DNA-binding capacity and transcriptional regulatory function. Zinc is essential for the structural stability of zinc-finger domains (Gao et al., 2023). The observed clinical improvement (reduced infection frequency and milder episodes) after zinc supplementation raises the possibility that the p. Arg218Trp variant partially impairs zinc coordination in the N-ZF domain, and supplementation may help stabilize residual protein function (Figure 4). However, this remains speculative without functional studies, and the benefit may be coincidental or nonspecific.

## 4 Limitations

This report is based on a single case, limiting generalizability. The observed improvements may be patient-specific or influenced by concurrent rehabilitation therapy and natural disease course. Whether zinc supplementation is beneficial in other patients with similar variants remains an open question requiring further investigation through larger cohorts and functional studies.

## 5 Conclusion

This case report describes a patient with a *de novo* missense variant in the N-terminal zinc-finger domain of *ZEB2* who presented with neurodevelopmental delay, atypical facial dysmorphism, and recurrent pulmonary infections, with a noted reduction in infections during zinc supplementation. These observations raise questions about potential associations with *ZEB2* variants and zinc-related mechanisms. Additional clinical cases and functional studies are needed to investigate these possibilities.

## Data Availability

The original contributions presented in the study are included in the article/supplementary material, further inquiries can be directed to the corresponding authors.
